# Pathological Technique

**Published:** 1898-10

**Authors:** 


PATHOLOGICAL TECHNIQUE.—A Practical Manual for the Pathological Laboratory, by Frank Burr Mallory, A. M. M. D., Assistant Professor of Pathology, Harvard Medical School, etc., and James Horner Wright, A. M., M. D., Director of the Laboratory of the Massachusetts General Hospital, etc. Published by W. B. Saunders, Philadelphia. Pages, 397. Price, cloth, $2.50.

This book is designed especially for practical use in pathological laboratories, especially for beginners, but, is nevertheless the best book for the general practitioner who does practical work, that we have yet seen. Usually the practitioner has to cull his entire library to find the information which is contained in this one volume.

The simplest methods that are used are given preference over the more complicated, and any physician who has a microscope of moderate power, and a very few accessories, with a case of post mortem instruments, can be his own pathologist. The subject is considered under three main heads: Post Mortem Examinations, Bacteriological Examinations, and Histological Methods. The only criticism that we are able to make is that more attention might have been profitably given to the chemistry of the subject. The work is thoroughly indexed, and is a valuable addition to our literature. J.

———

ORTHOPÆDIC SURGERY.—By James E. Moore, M. D., Professor of Orthopædic and Clinical Surgery in the College of Medicine of the University of Minnesota, etc. Illustrated. Published by W. B. Saunders, Philadelphia, 1898. Pages, 352. Price, cloth, $2.50.

This is a text book for students and practitioners, but as the author has excluded all methods of diagnosis and treatment, except such as he has found of value, it is essentially clinical, though quite complete. Orthopædia is essentially an American science, and rich contributions have been made to it from the rich storehouse of American inventive genius. In these days of national spirit, not to say national exultation, our profession should not fail to claim its own, lest haply we shall be considered as having nothing to claim.

The subject is considered in twenty-four chapters conveniently divided and indexed, making access to its contents easy. Much attention has been given to the early diagnosis of deformities, and if this is done simple treatment and appliances are wonderfully successful. The work fairly represents the present status of the science, and shows over the complicated methods and appliances, formerly in use. J.

